# Probiotic *Lactocaseibacillus casei* NK1 Enhances Growth and Gut Microbiota in Avian Pathogenic *Escherichia coli* Challenged Broilers

**DOI:** 10.3390/ani15081136

**Published:** 2025-04-15

**Authors:** Nimra Khalid, Syed Mohsin Bukhari, Waqas Ali, Ali Ahmad Sheikh, Hafiz Muhammad Abdullah, Ali Nazmi

**Affiliations:** 1 Department of Animal Sciences, College of Food Agriculture and Environmental Sciences, The Ohio State University, Wooster, OH 44691, USA; khalid.78@osu.edu (N.K.); abdullah.97@osu.edu (H.M.A.); 2 Department of Wildlife and Ecology, University of Veterinary and Animal Sciences, Lahore 54000, Pakistan; mohsin.bukhari@uvas.edu.pk (S.M.B.); waqas.ali@uvas.edu.pk (W.A.); 3 Institute of Microbiology, University of Veterinary and Animal Sciences, Lahore 54000, Pakistan; ali.ahmad@uvas.edu.pk; 4 Food for Health Discovery Theme, The Ohio State University, Columbus, OH 43210, USA

**Keywords:** *Escherichia coli*, *Lactocaseibacillus casei* NK1, gut microbiota, growth performance, broiler

## Abstract

The present study was conducted to assess the efficacy of Laboratory-Isolated *Lactocaseibacillus casei* NK1 (Lc. NK1) in broilers hypothesizing that, Lc. NK1 supplementation will enhance growth performance, modulate the gut microbiome, and reduce fecal pathogenic *Escherichia coli* in broilers. The experiment spanned 35 days where 60 one-day-old broiler chicks were randomly assigned to four treatment groups (*n* = 15); control-group with no treatment (NC), APEC (challenged with *E. coli* only), CProb (received commercial probiotics), and LNK1 (treated with Lc. NK1). The APEC, CProb, and LNK1 groups were infected with *E. coli* O78 strain at 11 days of age. Growth performance analysis revealed that the LNK1 group by day 35 gained body weight similar to the CProb group, with both groups significantly outperforming the APEC group (*p* < 0.001). Both the LNK1 and CProp groups exhibited similar reduction in *E. coli* while increasing *Lactobacillus* colorizations in the cloacal swabs from day 21 to 35 of age (*p* > 0.05). Metagenomic analysis using 16S rRNA sequencing showed that the LNK1 group maintained a diverse and balanced gut microbiota, characterized by increased Firmicutes and reduced Proteobacteria. In contrast, the APEC group exhibited reduced diversity and dominance of *Escherichia-Shigella* (*p* < 0.001). These findings suggest Lc. NK1 could be a promising probiotic for enhancing gut health and growth performance in broilers, even under pathogenic challenges, offering a potential alternative to antibiotics in poultry production.

## Simple Summary

This study tested the potential of a newly isolated *Lactocaseibacillus casei* NK1 (Lc. NK1) strain as a probiotic to enhance the growth and health of broiler chickens during the Avian Pathogenic *Escherichia coli* (APEC) challenge. The Lc. NK1 treated chickens achieved comparable growth performance to the birds that received a standard probiotic. The results suggest that Lc. NK1 inhibits the harmful bacteria while promoting beneficial gut microbiota offering a natural way to improve poultry health and growth.

## 1. Introduction

Avian Pathogenic *Escherichia coli* (APEC) is a major bacterial-pathogen that causes colibacillosis, an acute and systemic disease affecting various poultry species, including chickens, turkeys, and ducks [1]. The impact of APEC on the poultry industry is substantial, leading to significant economic losses worldwide. It is one of the leading causes of mortality in poultry, with rates reaching up to 20%. Additionally, APEC infections result in a 2% decline in live weight and a 2.7% reduction in feed conversion ratio, negatively affecting overall growth performance [2,3]. APEC causes both local and systemic infections, including perihepatitis, airsacculitis, pericarditis, egg peritonitis, omphalitis, cellulitis, and osteomyelitis/arthritis [4,5]. Globally, the three most prevalent APEC serotypes O78, O2, and O1 account for over 80% of cases [5,6].

The overuse and misuse of antibiotics have led to the emergence of antibiotic resistant bacteria posing significant challenges to both poultry health and public health [7]. In response to the growing threat of antibiotic resistance, many countries and regulatory bodies have implemented policies and restrictions on the use of antibiotics as growth-promoters (AGPs) in poultry production [8,9]. Therefore, there is an urgent need for novel alternatives to AGPs to mitigate poultry diseases and to enhance animal health.

Probiotics, particularly *Lactobacillus* strains, are emerging as promising alternatives to antibiotics in poultry production due to their ability to competitively exclude pathogens such as *Salmonella* [10], *Escherichia coli* (*E. coli*) [5,11], and *Campylobacter* [12]. They exert beneficial effects through multiple mechanisms, including the production of antimicrobial compounds, competition for adhesion sites, and modulation of host immune responses [13,14,15,16,17,18].

Previously, many researchers have isolated different strains of *Lactobacillus casei* (renamed as *Lactocaseibacillus casei* (*L. casei*) in 2020) and described its potential effects, such as improving lipid profiles, exerting antihypertensive and anti-hyperglycemic effects, and enhancing the quality of both dairy and non-dairy products [19,20,21]. Various research on *Lactobacillus* strains in poultry has largely focused on in vitro evaluations; for instance, *L. casei* FBL6 has shown tolerance to acid and bile salts, inhibitory activity against pathogenic bacteria, and antioxidant properties, highlighting its robust probiotic potential [22]. *L. farciminis* LN19 and *L. acidophilus* demonstrated activity against *E. coli* O78, but without in vivo validation [23,24]. In Pakistan, *L. casei* SABA6 showed activity against multidrug-resistant *E. coli* in rabbits [25], and dairy isolates like *L. paracasei* and *L. fermentum* have not been tested for antagonistic activity against pathogens [26]. Commercial probiotics used in poultry commonly include *L. plantarum*, *L. rhamnosus*, and *L. acidophilus* [27].

In our laboratory, we previously isolated a novel *L. casei* strain, Lc. NK1, from healthy broiler gut and confirmed it through 16s rRNA sequencing. This strain demonstrated comparable in vitro probiotic properties, including antagonistic activity against avian pathogenic *E. coli*. Therefore, this study was designed to further explore the efficacy of Lc. NK1, hypothesizing that its supplementation will improve growth performance, modulate the gut microbiome, and reduce APEC colonization in broiler chickens.

## 2. Materials and Methods

### 2.1. Ethical Approval

This study adhered to the ethical principles and guidelines set forth by the University of Veterinary and Animal Sciences Lahore Pakistan’s Ethical Review Committee, with reference number DR/780, approved on 21 December 2022.

### 2.2. Bacterial Strains and Culturing

Lc. NK1 (Accession no: PP831161) used in this study was isolated from the ileum of healthy broilers [28]. Lc. NK1 was grown overnight in de Man, Rogosa, and Sharpe (MRS) broth (Gibco, Carlsbad, CA, USA) at 37 °C under microaerophilic conditions. A pathogenic *E. coli* O78 (APEC) strain, obtained from the Veterinary Research & Disease Investigation Center, Abbottabad, Pakistan, was incubated in Luria–Bertani (LB) medium at 37 °C for 24 h under aerobic condition. Bacterial suspensions of 10^8^ CFU/mL for Lc. NK1 and 10^5^ CFU/mL for *E. coli* O78 were prepared and adjusted using a spectrophotometer.

### 2.3. Experimental Design and Chicken Rearing

This study followed a completely randomized design (CRD) to evaluate the impact of APEC infection and probiotic interventions on broiler growth performance and microbiome composition. A total of 60 one-day-old Ross 308 broiler chicks were procured from a local hatchery and reared at a poultry farm in Okara, Pakistan. The birds were housed in floor pens with rice husk as bedding and were given ad libitum access to water and feed throughout the 35-day trial period. The ingredient composition and calculated nutrient content of the experimental diets fed during different feeding phases (days) are presented as Supplementary File in Table S1. Chicks were randomly assigned to one of four experimental groups (NC, APEC, CProb, and LNK1), with 15 birds per group housed in separate pens. Nutrient requirements were maintained according to NRC guidelines [29]. The NC group was maintained without any challenge or treatment. The APEC group was challenged with *E. coli* O78 with no treatment. The CProb group was supplemented with a commercial probiotic Protexin (Hilton Pharma Ltd., Karachi, Pakistan) 1 g/liter (10^7^ CFU/mL) in the drinking water, according to manufacture recommendation. The LNK1 group was orally administered 10^8^ CFU/mL MRS containing Lc. NK1 on days 1, 7, 14, 21, and 28 of age. At day 11 of age, all groups, except the NC, were challenged with *E. coli* O78 at a dose of 10^5^ CFU/mL PBS per bird. Mortality was observed throughout the experiment.

### 2.4. Microbial Count

For microbial counting, cloacal swabs of three randomly selected chickens from each treatment group were plated weekly. Sterile mini-tip swab was inserted in the cloaca, rotated five times, and rolled to coat with fecal materials. Swabs were placed in sterile containers with 1 mL of sterile PBS on ice. The samples were serially diluted in PBS and plated on MacConkey agar with Congo red dye (3% *v*/*v*) for APEC [30] and MRS agar for *Lactobacillus* enumeration.

### 2.5. Body Weight Gain

All birds were weighed weekly on days 0, 7, 14, 21, 28, and 35 of age. Body weight gain (BWG) for each interval (0–7, 7–14, 14–21, 21–28, and 28–35 days) was calculated as the difference between the initial and final weights for the respective period, with day 0 representing the baseline weight of one-day-old chicks.

### 2.6. Metagenomic Analysis and Microbial Diversity Assessment Using 16S rRNA Sequencing

#### 2.6.1. Cecal Sample Collection

At 35 days of age, 5 birds per group (APEC and LNK1) were euthanized by cervical dislocation and cecal contents were aseptically collected, pooled and homogenized to represent the microbial composition of the entire group [31] following the method outlined by Stanley et al. [32]. The NC group was not assessed for microbial diversity because it served as a baseline for healthy, untreated conditions, and the broiler gut microbiota under such conditions is already well-documented [33,34]. Similarly, the CProb group was excluded because it represents a commercially available product and not the study’s experimental innovation.

#### 2.6.2. Genomic DNA Isolation

Genomic DNA was isolated from cecal contents using the CTAB (Cetyl trimethylammonium bromide) method. For this, 200 mg of homogenized cecal content was lysed in CTAB extraction buffer with 20% SDS and 20 µL of Proteinase K (10 mg/mL) and incubated at 65 °C for 3 h. Phase separation was performed using the chloroform: isoamyl alcohol (24:1) method. DNA was precipitated using 0.6 volumes of isopropanol at −20 °C for 30 min and then washed with 70% ethanol. The final suspension was prepared in nuclease-free water following RNase A treatment. The DNA was then suspended in double deionized water, and its concentration and purity were assessed using a Nanodrop 2000™ and agarose gel electrophoresis, respectively.

#### 2.6.3. 16S rRNA Gene Amplification and Sequencing

The 16S rRNA genes were amplified using the 16S hyper-variable V4 region using the forward primer 515F (5′-GTGCCAGCMGCCGCGGTAA-3′) and the reverse primer 806R (5′-GGACTACHVGGGTWTCTAAT-3′) [35,36], with barcodes for multiplexing. Libraries were prepared following the MiSeq Reagent Kit Preparation Guide (Illumina, San Diego, CA, USA). The library concentrations were quantified using Q-PCR and Qubit, and sequencing was performed on an Illumina Mi-Seq sequencer using the paired-end method. The resulting raw sequencing data were stored in FASTQ format.

#### 2.6.4. 16S rRNA Sequence Processing and Analysis

Initial processing of the raw FASTQ files begin with quality control using FastQC [37]. Subsequently, the reads were trimmed using Trim Galore (https://www.bioinformatics.babraham.ac.uk/projects/trim_galore/, accessed on 30 July 2024), a tool that combines Cutadapt (https://cutadapt.readthedocs.io/en/stable/, accessed on 30 July 2024) and FastQC to remove adapters and improve quality. After trimming, FastQC was used again to assess the quality of the trimmed reads.

After quality filtering and trimming, taxonomic classification was performed using Kraken2 (https://ccb.jhu.edu/software/kraken2/, accessed on 30 July 2024) with the Kraken2 Standard Database, which includes bacterial, archaeal, and viral genomes from RefSeq [38]. Bracken was then used to estimate relative abundances at the genus level using the -G flag, and taxonomy assignment was guided using SILVA references [39,40] for reliable genus-level identification. Only sequences with read counts above a threshold of 10 were considered, and percentages were recalculated for these retained levels.

### 2.7. Statistical Analysis

Data analysis was performed using IBM SPSS Statistics 28 and GraphPad PRISM v10.0.3 (GraphPad, Boston, MA, USA). Normality and ANOVA assumptions were checked prior to analysis. One-way ANOVA was conducted to evaluate the effect of treatments on microbial count data and weight gain in chickens, followed by post hoc Tukey HSD tests for mean separation. Survival curves were constructed using the Kaplan–Meier method in GraphPad PRISM, and statistical significance was assessed using the log-rank test in SPSS. Differential genus-level abundance was assessed using the Wilcoxon rank-sum test in STAMP (Statistical Analysis of Metagenomic Profiles). A *p*-value of <0.05 was considered statistically significant.

## 3. Results

### 3.1. Body Weight Gain

**Figure 1:**
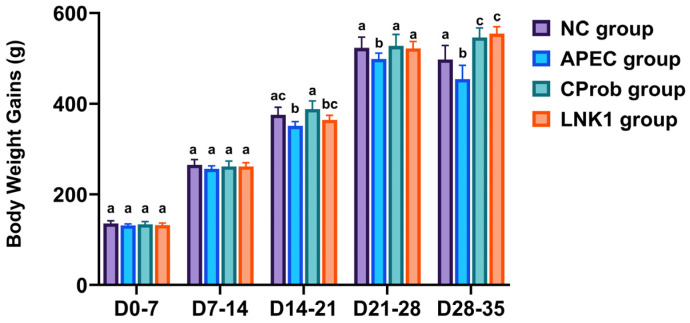
Effect of *Lactobacillus* supplementation and APEC infection on BWGs. This bar graph shows the BWG of chickens at different intervals (D0–7, D7–14, D14–21, D21–28, D28–35) across four experimental groups: NC, APEC, CProb, and LNK1. Statistical differences between groups at each time point, indicated by different letters above the bars, were determined using one-way ANOVA and Tukey HSD tests. Bars represent mean ± SEM.

Until day 14 of age, there were no differences between groups in BWGs (Figure 1). The BWG of LNK1 group was comparable to NC and APEC groups at day 21. By day 35, LNK1 exhibited significantly higher BWG than the NC and APEC groups. Interestingly, Both LNK1 and CProb groups maintained similar BWGs at days 28 and 35.

### 3.2. Mortality

**Figure 2:**
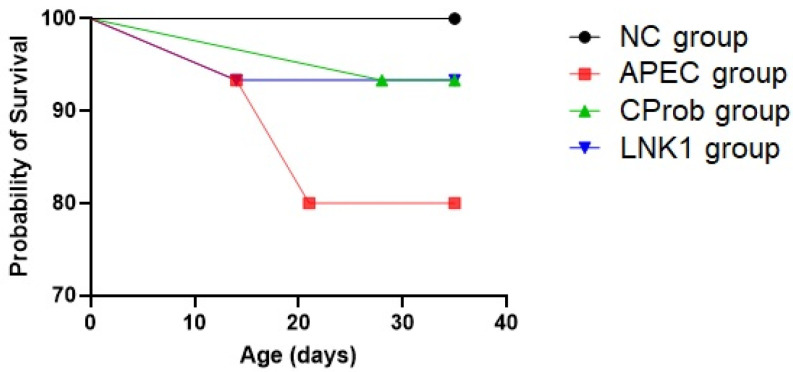
Survival curves for broilers across different treatment groups. The Kaplan–Meier survival curves illustrate the survival probabilities of NC, APEC, CProb and LNK1 groups.

Following bacterial challenge at day 11 of age, LNK1 group and CProb groups had less mortality (6.7%) compared to the APEC group (20%) (Figure 2). No mortality was observed in the NC group. Although, the mortality rate was not statistically significant (*p* = 0.244) between groups, these results suggest a trend where the CProb and LNK1 treatments may reduce APEC-associated mortality compared to the untreated APEC group.

### 3.3. Microbial Count in Cloacal Swabs

**Figure 3:**
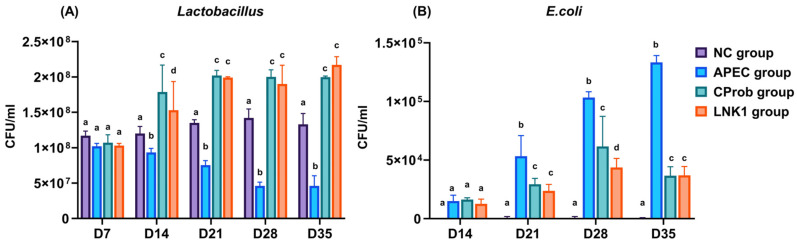
*Lactobacillus* and APEC counts in cloacal swabs at various time points. (**A**) *Lactobacillus* counts at days 7, 14, 21, 28, and 35 of age. (**B**) *E. coli* counts at days 14, 21, 28, and 35 of age. Statistical differences between groups at each time point, indicated by different letters above the bars, were determined using one-way ANOVA and Tukey HSD tests. Bars represent mean ± SEM.

At 14th day of age, both LNK1 and CProb groups exhibited significant increases in *Lactobacillus* counts in their cloacal swabs, and they maintained these increases to day 35 (Figure 3A). The increase in *Lactobacillus* counts coincided with a reduction of *E. coli* counts in the LNK1 and CProb groups at 21 to 35 days of age, (Figure 3B), highlighting their potential effectiveness in mitigating APEC colonization.

### 3.4. Metagenomic Analysis Using 16S rRNA Sequencing

In the sequencing run, APEC group produced 150,190 reads, while LNK1 group generated 196,568 reads.

#### 3.4.1. The Alpha Diversity Metrics

The alpha diversity metrics for the gut microbiota of LNK1 and APEC groups, present a contrasting picture of microbial health and diversity (Table 1). The LNK1 group demonstrates a balanced microbial environment with moderate diversity levels, as indicated by the Shannon index of 0.935 and the Simpson’s Reciprocal Index of 1.885.

Conversely, the APEC group exhibits extremely low diversity, with the Shannon index of only 0.026 and the Simpson’s index of 0.006, indicating a microbial community dominated almost entirely by one or a few species. This is further supported by the Berger–Parker index nearing 1 (0.997), implying that nearly the entire microbial population is comprised of a single dominant species.

#### 3.4.2. Microbial Diversity and Community Structure Analysis

**Figure 4:**
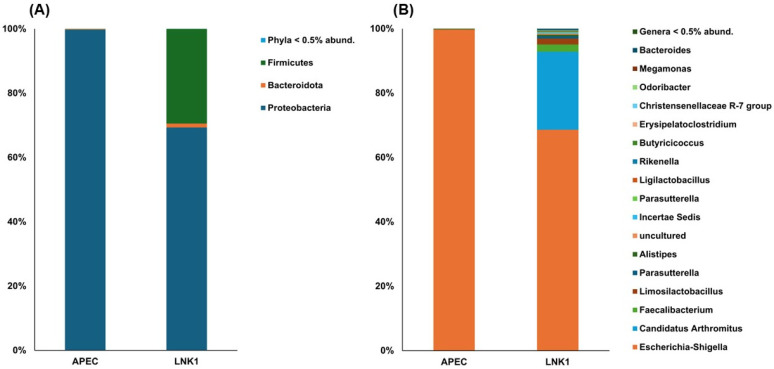
Community analysis of gut microbiota of APEC and LNK1 groups at the phylum (**A**) and genera (**B**) level.

Community structure analysis results revealed markedly variations between the APEC and LNK1 groups, both at phylum and genera levels (Figure 4). At the phylum level, the APEC group was predominantly characterized by Proteobacteria, suggesting a less diverse microbial environment, while the LNK1 group displayed a healthier, more varied composition with substantial representation of Firmicutes, Bacteroidetes and Proteobacteria (Figure 4A). At the genera level (Figure 4B), the APEC group was dominated by *Escherichia-Shigella*, indicative of a pathogen-rich environment, whereas the LNK1 group showed a wider range of beneficial microbes, including *Lactobacillus* and Bacteroides, which are associated with a more balanced and healthier gut microbiota.

#### 3.4.3. Relative Abundance Comparison

**Figure 5:**
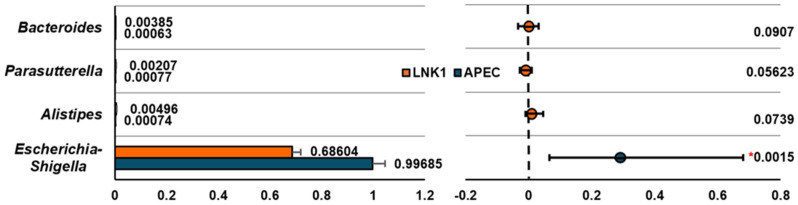
Extended error bar plot for relative abundance of bacterial genera between the LNK1 (orange) and APEC (blue) groups. Statistical differences were assessed using the Wilcoxon rank-sum test in STAMP. Mean relative abundance and *p*-values are displayed for each genus; *p* < 0.05 was considered statistically significant. * Indicates a significant difference at *p* < 0.05.

The comparative analysis revealed that *Escherichia-Shigella* significantly dominates in the APEC group compared to LNK1 (*p* < 0.05), while *Bacteroides*, *Parabacteroides*, and *Alistipes* are more prevalent in LNK1, although these differences were not significant as shown in Figure 5.

## 4. Discussion

In this study, we investigated the in vivo efficacy of the newly isolated Lc. NK1 strain in broiler chickens focusing on its potential to enhance growth performance, reduce APEC colonization and modulate the gut microbiome. Previously, *Lactobacillus* strains were isolated from the ileum of both colibacillosis diseased and healthy broilers [28], and their competitive exclusion potential against *E. coli* O78 was assessed. Among these isolates, Lc. NK1, obtained from healthy broilers, demonstrated strong in vitro antagonistic activity against lab isolated APEC and pathogenic *E. coli* O78 strains.

The current results demonstrate that, Lc. NK1 supplementation positively influenced body weight gain (BWG) in broilers similar to that of the commercial probiotics (CProb), particularly in later growth stages. This improvement in BWG suggests that, Lc. NK1 may exert beneficial actions that extend beyond antimicrobial activity, possibly influencing nutrient absorption and overall gut health. Previous studies have shown that *Lactobacillus* strains can improve feed conversion ratios and nutrient utilization by modifying the gut microbiota composition, thereby optimizing the digestive process and enhancing growth performance [41,42]. These effects are often linked to the production of enzymes that aid in the breakdown of feed and increase nutrient availability [42]. While some studies suggest potential immunomodulatory roles of probiotics no such effects were accessed directly in present study.

Moreover, the robust BWG observed in the LNK1 group, despite high APEC counts, may be partially attributed to potential immunomodulatory effects, although such effects were not directly measured in this study. *Lactobacillus* strains are known to enhance the intestinal barrier function and stimulate anti-inflammatory cytokines, which can help mitigate the impact of infections on growth performance [43]. This immune modulation can lead to a more resilient physiological state that allows for better growth even under pathogenic stress [44]. These findings were supported by the study of Jha et al. who demonstrated that dietary probiotics could lead to improvements in growth metrics by enhancing gut health and immune function, thereby allowing animals to grow efficiently even under pathogenic pressure [45].

In the microbial assessment of cloacal swabs, the LNK1 group exhibited a reduction in *E. coli* CFU comparable to the CProb group, likely due to competitive exclusion and mucosal barrier reinforcement. Probiotics, including Lc. NK1, enhance gut health by competing with pathogens for nutrients and adhesion sites on the intestinal epithelium, thereby inhibiting *E. coli* colonization and proliferation [46]. Moreover, the significantly higher *Lactobacillus* counts in the LNK1 group and CProb group suggest robust colonization and activity within the gut. Previous findings have shown similar trends where probiotic administration in poultry resulted in decreased levels of gut pathogens and increased populations of beneficial microbes [47,48]. Notably, the commercial probiotic used in this study is a multi-strain formulation, whereas Lc. NK1 is a single-strain isolate. While Lc. NK1 did not outperform the commercial product, its comparable efficacy highlights its potential as an alternative probiotic candidate. Future research should investigate its compatibility with other strains to assess its potential inclusion in multi-strain probiotic consortia. In addition, dietary inclusion of *Lactobacillus*-based probiotics led to reduced *Salmonella* counts in broilers [48], which, aligns with our findings regarding reduction in *E. coli*. These studies collectively underscore the potential of probiotics to enhance gut health through a combination of microbial antagonism, competitive exclusion, and immune modulation.

Gut microbial diversity was analyzed between the APEC group and LNK1 group to identify the core functional differences caused by the probiotic. The alpha diversity metrics revealed distinct differences, with the LNK1 group consistently showing higher diversity indices across all measures suggests a relatively healthy gut with no single species dominating excessively. Such diversity is typically observed in well-managed probiotic interventions and is crucial for maintaining gut ecosystem stability. Higher Shannon and Simpson indices in the LNK1 group indicate greater species richness and evenness, which are linked to improved gut integrity, immune modulation, and nutrient absorption. The elevated Fisher’s index in LNK1 further supports the presence of a rich and resilient microbiome, whereas the lower values in the APEC group, along with a near-maximal Berger–Parker index, point to microbial dominance and dysbiosis [49], characterized by a dominance of the Proteobacteria phylum, a major group of Gram-negative bacteria that includes both pathogenic and non-pathogenic species. A healthy poultry gut microbiota is generally associated with a balanced Firmicutes-to-Bacteroidetes ratio [50], and disruptions in this balance, especially an increase in Proteobacteria, have been linked to gut inflammation and adverse health outcomes in various hosts [51,52].

The LNK1 group exhibited a balanced microbial diversity, including Proteobacteria, Bacteroidetes, and Firmicutes, indicating that, Lc. NK1 effectively suppressed pathogenic bacteria while enhancing beneficial bacterial diversity. This balance between Bacteroidetes and Firmicutes is crucial for nutrient absorption, energy harvest, and overall health [53]. Compared to the APEC group, the LNK1 group was colonized with a healthier microbial composition, consistent with literature linking optimal phyla ratios to improved feed efficiency and reduced gastrointestinal diseases in broilers [49,54].

The increase in Bacteroidetes likely reflects *Lactobacillus*’s ability to create a favorable gut environment, supporting beneficial bacteria that compete against pathogens [55]. *Bacteroides* play a key role in breaking down complex carbohydrates into short-chain fatty acids (SCFAs), including butyrate, acetate, and propionate. These SCFAs support colonic health by providing energy for colonocytes and exerting anti-inflammatory effects, contributing to overall gut health [56]. Additionally, the presence of *Faecalibacterium*, known for anti-inflammatory properties and gut barrier support, suggests enhanced SCFA production and immune modulation in the LNK1 group, promoting superior gut health in broilers [57].

Although the control group in this study was not assessed for gut microbial diversity, previous research consistently highlights the dominance of Firmicutes (40–60%) and Bacteroidetes (20–40%) as key contributors to energy metabolism and gut health, with minor contributions from Proteobacteria (5–10%) and Actinobacteria (3–8%) [34,58,59]. In contrast, the LNK1 group displayed a distinct microbial profile, suggesting that, Lc. NK1 can thrive and sustain its growth in a Proteobacteria-dominant environment while promoting microbial diversity and gut health. This is further supported by the observed improvements in weight gain and microbial count results, underscoring the efficacy of LNK1 in counteracting gut dysbiosis and promoting a balanced microbiota.

Additionally, the LNK1 group harbored unique taxa such as Christensenellaceae R-7, known for its role in promoting gut health, enhancing microbial diversity, and supporting lipid metabolism [60,61]. This taxon may complement *Lactobacillus* in optimizing lipid metabolism and improving overall host health. Further investigation is required to evaluate its synergistic effects with other probiotics, pathogen resistance, and cost-effectiveness for broader poultry applications.

While this study provides valuable insights into the impact of Lc. NK1 on growth performance and gut microbiota in broilers, some limitations should be acknowledged. This study was conducted over a relatively short-term period (35 days), which may not fully capture the long-term effects of probiotic supplementation on poultry health or microbial stability. Moreover, no histopathological examination of intestinal tissues was performed; such data could have strengthened the link between microbial shifts and intestinal development and health.

## 5. Conclusions

The findings suggest that *L. casei* NK1, originally isolated from the ileum of healthy broilers, demonstrated beneficial effects in this controlled study by promoting a healthier gut environment, increasing Firmicutes, and reducing Proteobacteria. These changes were associated with improved growth and resilience under pathogenic challenge. However, further studies are needed to assess its immunomodulatory potential and validate its commercial feasibility through large-scale field trials.

## Figures and Tables

**Table 1:** Comparison of alpha diversity metrics between LNK1 and APEC groups.

Metrics (Unitless)	LNK1	APEC
Shannon’s Diversity	0.935	0.026
Simpson’s Index of Diversity	0.47	0.006
Simpson’s Reciprocal Index	1.885	1.006
Berger-Parker’s Diversity	0.686	0.997
Fisher’s Index	1.438	0.389

## Data Availability

The sequencing data are available via NCBI’s Sequence Read Archive (SRA) under the BioProject number PRJNA1150550. The data can be accessed at https://www.ncbi.nlm.nih.gov/sra/PRJNA1150550, accessed on 21 August 2024.

## Supplementary Materials

The following supporting information can be downloaded at: https://www.mdpi.com/article/10.3390/ani15081136/s1, Table S1: Ingredient composition and calculated nutrient content of the experimental diets fed during different feeding phases (days).

## References

[1] Nolan L.K., Barnes H.J., Vaillancourt J.P., Abdul-Aziz T., Logue C.M. (2013). Colibacillosis. Diseases of Poultry.

[2] Dho-Moulin M., Fairbrother J.M. (1999). Avian pathogenic Escherichia coli (APEC). Vet. Res..

[3] Guabiraba R., Schouler C. (2015). Avian colibacillosis: Still many black holes. FEMS Microbiol. Lett..

[4] Mellata M. (2013). Human and avian extraintestinal pathogenic Escherichia coli: Infections, zoonotic risks, and antibiotic resistance trends. Foodborne Pathog. Dis..

[5] Kathayat D., Lokesh D., Ranjit S., Rajashekara G. (2021). Avian pathogenic Escherichia coli (APEC): An overview of virulence and pathogenesis factors, zoonotic potential, and control strategies. Pathogens.

[6] Thøfner I., Christensen J.P. (2021). Bacterial diseases in poultry. Advancements and Technologies in Pig and Poultry Bacterial Disease Control.

[7] Mehdi Y., Létourneau-Montminy M.P., Gaucher M.L., Chorfi Y., Suresh G., Rouissi T., Brar S.K., Côté C., Ramirez A.A., Godbout S. (2018). Use of antibiotics in broiler production: Global impacts and alternatives. Anim. Nutr..

[8] Martin M.J., Thottathil S.E., Newman T.B. (2015). Antibiotics overuse in animal agriculture: A call to action for health care providers. Am. J. Public Health.

[9] Andrew Selaledi L., Mohammed Hassan Z., Manyelo T.G., Mabelebele M. (2020). The current status of the alternative use to antibiotics in poultry production: An African perspective. Antibiotics.

[10] Tian C., Wang L., Liu M., Liu J., Qiu M., Chen Y. (2024). Isolation and Identification of Chicken-Derived Lactic Acid Bacteria: In Vitro Probiotic Properties and Antagonistic Effects against *Salmonella pullorum*, *Staphylococcus aureus*, and *Escherichia coli*. Microorganisms.

[11] Tsega K.T., Kagira J.M., Tessema N.B., Mekuria S.A. (2024). Effects of *Lactobacillus* probiotics supplemented with concentrate feed on growth performance, carcass characteristics, and caecal microflora of RIR chickens. Cogent. Food Agric..

[12] Wishna-Kadawarage R.N., Hickey R.M., Siwek M. (2024). In-vitro selection of lactic acid bacteria to combat *Salmonella enterica* and *Campylobacter jejuni* in broiler chickens. World J. Microbiol. Biotechnol..

[13] Jin L.Z., Marquardt R.R., Baidoo S.K. (2000). Inhibition of enterotoxigenic *Escherichia coli* K88, K99 and 987P by the *Lactobacillus* isolates from porcine intestine. J. Sci. Food Agric..

[14] Ehrmann M.A., Kurzak P., Bauer J., Vogel R.F. (2002). Characterization of lactobacilli towards their use as probiotic adjuncts in poultry. J. Appl. Microbiol..

[15] La Ragione R.M., Narbad A., Gasson M.J., Woodward M.J. (2004). In vivo characterization of *Lactobacillus johnsonii* FI9785 for use as a defined competitive exclusion agent against bacterial pathogens in poultry. Lett. Appl. Microbiol..

[16] Ismail S., Ajeng A.A., Ramli M.R., Ameen F., Nasir N., Lakshmikandan M. (2024). Comparison of novel bacillus salmalaya 139si and lactobacillus as probiotics in the drinking water of chicks. J. Anim. Plant Sci..

[17] Al-Hazmi N.E., Naguib D.M. (2024). Antioxidant and antibacterial activities of nano-probiotics versus free probiotics against gastrointestinal pathogenic bacteria. Indian J. Microbiol..

[18] Idebi J.A., Akeredolu O.S., Omotosho T. (2024). Isolation and Molecular Identification of Lactic Acid Bacteria from Fermented Maize Grain (Ogi) and their Antimicrobial Activities against Pathogenic Bacteria. IJIRMPS.

[19] Zheng J., Wittouck S., Salvetti E., Franz C.M., Harris H.M., Mattarelli P., O’toole P.W., Pot B., Vandamme P., Walter J. (2020). A Taxonomic Note on the Genus Lactobacillus: Description of 23 Novel Genera, Emended Description of the Genus *Lactobacillus* Beijerinck 1901, and Union of Lactobacillaceae and Leuconostocaceae. Int. J. Syst. Evol. Microbiol..

[20] Yin D., Du E., Yuan J., Gao J., Wang Y., Aggrey S.E., Guo Y. (2017). Supplemental thymol and carvacrol increases ileum *Lactobacillus* population and reduces effect of necrotic enteritis caused by Clostridium perfringes in chickens. Sci. Rep..

[21] Pimentel T.C., Brandão L.R., de Oliveira M.P., da Costa W.K.A., Magnani M. (2021). Health benefits and technological effects of Lacticaseibacillus casei-01: An overview of the scientific literature. Trends Food Sci. Technol..

[22] Kim E., Yang S.M., Kim D., Kim H.Y. (2022). Complete genome sequencing and comparative genomics of three potential probiotic strains, *Lacticaseibacillus casei* FBL6, *Lacticaseibacillus chiayiensis* FBL7, and *Lacticaseibacillus zeae* FBL8. Front. Microbiol..

[23] Thuy N.P., Trai N.N. (2024). Screening of *Lactobacillus* from Noi chicken gut as potential probiotics against poultry pathogens. Biodivers. J. Biol. Divers..

[24] Su S.S., Wang Y.J., Qi H.T., Wang L., Huang Z., Chen J.D., Zhang G.H. (2012). Effect of cell wall extracts from *Lactobacillus acidophilus* on pathogenic *E. coli* O78 binding to chicken intestinal brush border membranes. Chin. J. Prev. Vet. Med..

[25] Fayyaz I., Zahoor M.A., Shahid M., Rasool M.H., Nawaz Z. (2018). Effect of *Lactobacillus casei* on serum interleukins following enteropathogenic *E. coli* infection in experimental rabbits. Pak. J. Pharm. Sci..

[26] Kabir S., Shahid M., Waseem M., Muzammil S., Nawaz Z., Rasool M.H., Saqalein M. (2020). Dairy origin *Lactobacilli*: Functional analyses and antagonistic potential against multidrug-resistant foodborne pathogens. Int. Food Res. J..

[27] Abbas G., Asif-Iqbal M., Riaz M., Sajid M., Zahid O., Wasim-Abbas S.Y., Zohaib M. (2018). Comparative effect of different levels of probiotics (Protexin) on hemato-chemical profile in broilers. Adv. Zool. Bot..

[28] Khalid N., Bukhari S.M., Ali W., Sheikh A.A. (2023). Comparative Study on the Predominance of *Lactobacillus* spp. and Escherichia Coli in Healthy vs Colibacillosis Diseased Broilers. Braz. J. Poult. Sci..

[29] National Research Council, Subcommittee on Poultry Nutrition (1994). Nutrient Requirements of Poultry.

[30] Berkhoff H.A., Vinal A.C. (1986). Congo red medium to distinguish between invasive and non-invasive *Escherichia coli* pathogenic for poultry. Avian Dis..

[31] Weinroth M.D., Belk A.D., Dean C., Noyes N., Dittoe D.K., Rothrock M.J., Ricke S.C., Myer P.R., Henniger M.T., Ramírez G.A. (2022). Considerations and best practices in animal science 16S ribosomal RNA gene sequencing microbiome studies. J. Anim. Sci..

[32] Stanley D., Geier M.S., Chen H., Hughes R.J., Moore R.J. (2015). Comparison of fecal and cecal microbiotas reveals qualitative similarities but quantitative differences. BMC Microbiol..

[33] Mancabelli L., Ferrario C., Milani C., Mangifesta M., Turroni F., Duranti S., Lugli G.A., Viappiani A., Ossiprandi M.C., van Sinderen D. (2016). Insights into the Biodiversity of the Gut Microbiota of Broiler Chickens. Environ. Microbiol..

[34] Rashid Z., Yousaf M.Z., Gilani S.M.H., Zehra S., Ali A., Azhar A., Galani S. (2021). Comparative Analysis of Chicken Cecal Microbial Diversity and Taxonomic Composition in Response to Dietary Variation Using 16S rRNA Amplicon Sequencing. Mol. Biol. Rep..

[35] Liu P.Y., Wu W.K., Chen C.C., Panyod S., Sheen L.Y., Wu M.S. (2020). Evaluation of compatibility of 16S rRNA V3V4 and V4 amplicon libraries for clinical microbiome profiling. bioRxiv.

[36] Katiraei S., Anvar Y., Hoving L., Berbée J.F., van Harmelen V., Willems van Dijk K. (2022). Evaluation of full-length versus V4-region 16S rRNA sequencing for phylogenetic analysis of mouse intestinal microbiota after a dietary intervention. Curr. Microbiol..

[37] Brown J., Pirrung M., McCue L.A. (2017). FQC Dashboard: Integrates FastQC results into a web-based, interactive, and extensible FASTQ quality control tool. Bioinformatics.

[38] Lu J., Salzberg S.L. (2020). Ultrafast and accurate 16S rRNA microbial community analysis using Kraken 2. Microbiome.

[39] Quast C., Pruesse E., Yilmaz P., Gerken J., Schweer T., Yarza P., Peplies J., Glöckner F.O. (2012). The SILVA ribosomal RNA gene database project: Improved data processing and web-based tools. Nucleic Acids Res..

[40] Ondov B.D., Bergman N.H., Phillippy A.M. (2011). Interactive metagenomic visualization in a Web browser. BMC Bioinform..

[41] Gao Q., Wang Y., Li J., Bai G., Liu L., Zhong R., Ma T., Pan H., Zhang H. (2022). Supplementation of multi-enzymes alone or combined with inactivated *Lactobacillus* benefits growth performance and gut microbiota in broilers fed wheat diets. Front. Microbiol..

[42] Yang J., Wang C., Huang K., Zhang M., Wang J., Pan X. (2020). Compound *Lactobacillus* sp. administration ameliorates stress and body growth through gut microbiota optimization on weaning piglets. Appl. Microbiol. Biotechnol..

[43] Vasquez R., Oh J.K., Song J.H., Kang D.K. (2022). Gut microbiome-produced metabolites in pigs: A review on their biological functions and the influence of probiotics. J. Anim. Sci. Technol..

[44] Abd El-Hack M.E., El-Saadony M.T., Alqhtani A.H., Swelum A.A., Salem H.M., Elbestawy A.R., Noreldin A.E., Babalghith A.O., Khafaga A.F., Hassan M.I. (2022). The relationship among avian influenza, gut microbiota and chicken immunity: An updated overview. Poult. Sci..

[45] Jha R., Das R., Oak S., Mishra P. (2020). Probiotics (direct-fed microbials) in poultry nutrition and their effects on nutrient utilization, growth and laying performance, and gut health: A systematic review. Animals.

[46] Callaway T.R., Edrington T.S., Anderson R.C., Harvey R.B., Genovese K.J., Kennedy C.N., Venn D.W., Nisbet D.J. (2008). Probiotics, prebiotics and competitive exclusion for prophylaxis against bacterial disease. Anim. Health Res. Rev..

[47] Fuller R. (2000). Probiotics in man and animals. J. Appl. Bacteriol..

[48] Higgins S.E., Higgins J.P., Wolfenden A.D., Henderson S.N., Torres-Rodriguez A., Tellez G., Hargis B. (2008). Evaluation of a Lactobacillus-based probiotic culture for the reduction of Salmonella enteritidis in neonatal broiler chicks. Poult. Sci..

[49] Bindari Y.R., Gerber P.F. (2022). Centennial Review: Factors affecting the chicken gastrointestinal microbial composition and their association with gut health and productive performance. Poult. Sci..

[50] Stojanov S., Berlec A., Štrukelj B. (2020). The influence of probiotics on the firmicutes/bacteroidetes ratio in the treatment of obesity and inflammatory bowel disease. Microorganisms.

[51] Looft T., Johnson T.A., Allen H.K., Bayles D.O., Alt D.P., Stedtfeld R.D., Sul W.J., Stedtfeld T.M., Chai B., Cole J.R. (2012). In-feed antibiotic effects on the swine intestinal microbiome. Proc. Natl. Acad. Sci. USA.

[52] Xing Z., Li H., Li M., Gao R., Guo C., Mi S. (2021). Disequilibrium in chicken gut microflora with avian colibacillosis is related to microenvironment damaged by antibiotics. Sci. Total Environ..

[53] Oakley B.B., Lillehoj H.S., Kogut M.H., Kim W.K., Maurer J.J., Pedroso A., Lee M.D., Collett S.R., Johnson T.J., Cox N.A. (2014). The chicken gastrointestinal microbiome. FEMS Microbiol. Lett..

[54] Makled M.N., Abouelezz K.F., Gad-Elkareem A.E., Sayed A.M. (2019). Comparative influence of dietary probiotic, yoghurt, and sodium butyrate on growth performance, intestinal microbiota, blood hematology, and immune response of meat-type chickens. Trop. Anim. Health Prod..

[55] Clavijo V., Flórez M.J. (2018). The gastrointestinal microbiome and its association with the control of pathogens in broiler chicken production: A review. Poult. Sci..

[56] Louis P., Flint H.J. (2017). Formation of propionate and butyrate by the human colonic microbiota. Environ. Microbiol..

[57] Sokol H., Pigneur B., Watterlot L., Lakhdari O., Bermúdez-Humarán L.G., Gratadoux J.J., Blugeon S., Bridonneau C., Furet J.P., Corthier G. (2008). *Faecalibacterium prausnitzii* is an anti-inflammatory commensal bacterium identified by gut microbiota analysis of Crohn disease patients. Proc. Natl. Acad. Sci. USA.

[58] Afridi O.K., Ali J., Chang J.H. (2020). Next-generation sequencing based gut resistome profiling of broiler chickens infected with multidrug-resistant Escherichia coli. Animals.

[59] Qi Z., Shi S., Tu J., Li S. (2019). Comparative metagenomic sequencing analysis of cecum microbiotal diversity and function in broilers and layers. 3 Biotech.

[60] Morotomi M., Nagai F., Watanabe Y. (2012). Description of Christensenella minuta gen. nov., sp. nov., isolated from human faeces, which forms a distinct branch in the order Clostridiales, and proposal of Christensenellaceae fam. Int. J. Syst. Evol. Microbiol..

[61] Wang G., Tang H., Zhang Y., Xiao X., Xia Y., Ai L. (2020). The intervention effects of *Lactobacillus casei* LC2W on *Escherichia coli* O157: H7-induced mouse colitis. Food Sci. Hum. Wellness.

