# Supplementary material for: Probiotic Lactocaseibacillus casei NK1 Enhances Growth and Gut Microbiota in Avian Pathogenic Escherichia coli Challenged Broilers
Source: Animals (Basel). 2025 Apr 15;15(8):1136. doi: 10.3390/ani15081136 (PMC12024338; doi:10.3390/ani15081136)
Supplement: Supplementary file 1 [file animals-15-01136-s001.zip › animals-3513956-supplementary.pdf]

Table: Ingredient composition and calculated nutrient content of the experimental diets fed during different feeding phases (days)

**Ingredients**	**Starter (0-7)**	**Grower (7-21)**	**Finisher (22-35)**
Maize (12% crude protein)	28.866	28.075	25.184
Rice Broken (12% crude protein)	25	26.471	32
Rice Polish (15% crude protein)	4	4	4
Soybean Meal (44.5% crude protein)	19.005	19.624	16.261
Canola Meal (34.5% crude protein)	8	5	4.5
Rapeseed Meal (36% crude protein)	2	2	1
Guar Meal (38% crude protein)	1.3	3	4
Sunflower Meal (26% crude protein)	2	1.5	1.5
Poultry Fat (Refined)	0.982	2.073	2.955
Limestone	0.843	0.884	0.693
Dicalcium Phosphate (18% Phosphorus)	0.342	0.441	0.339
Salt	0.184	0.192	0.179
Soda Bicarbonate	0.234	0.234	0.175
Lysine Sulphate	0.668	0.622	0.7
DL-Methionine	0.312	0.308	0.314
L Threonine	0.189	0.161	0.176
L Tryptophan	0.007	-	-
L Arginine	0.068	-	-
L Valine	0.051	0.046	0.071
L Isoleucine	0.119	0.089	0.114
L Glycerine	0.07	-	0.029
Phytase (10,000 FTU/g)	0.01	0.01	0.01
Vitamin Premix[1]	0.075	0.075	0.075
Mineral Premix[2]	0.075	0.075	0.075
Choline Chloride	0.1	0.12	0.15
Meat and Bone Meal (40% crude protein)	1.5	1	1
Poultry Meal (50% crude protein)	4	4	4.5
Total	100	100	100
**Calculated nutrient composition (% unless specified)**			
Dry Matter	88.99	89.13	89.27
Metabolizable Energy (kcal/kg)	2800	2900	3000
Crude Protein	21.61	21	20
Crude Fat (EE)	4.38	5.48	6.47
Crude Fiber	4.18	3.94	3.69
Calcium	0.95	0.9	0.8
Available Phosphorus	0.44	0.42	0.4
Digestible Lysine	1.23	1.17	1.13
Digestible Methionine	0.61	0.59	0.58
Methionine + Cystine	0.91	0.88	0.86
Digestible Threonine	0.84	0.79	0.76
Digestible Tryptophan	0.21	0.2	0.2
Digestible Arginine	1.35	1.3	1.25
Digestible Valine	0.91	0.89	0.86
Digestible Isoleucine	0.84	0.79	0.77
Sodium	0.18	0.18	0.16
Choline	918.29	791.88	688.22
Total NSP	2.27	2.21	1.98

[1] *Vitamin premix per kilogram of feed: 12,000 IU of vitamin A, 5,000 IU of vitamin D_3, 80 IU of vitamin E, and 3.2 mg of vitamin K. In addition, it contained 3.2 mg of thiamine (vitamin B_1), 8.6 mg of riboflavin (vitamin B_2), 65 mg of niacin, 20 mg of pantothenic acid, 4.3 mg of pyridoxine (vitamin B_6), 0.22 mg of biotin, 2.20 mg of folic acid, and 0.017 mg of vitamin B_{12}.*

[2] *Mineral premix per kilogram of feed: 16 mg of copper, 1.25 mg of iodine, 20 mg of iron, 120 mg of manganese, 0.30 mg of selenium, and 110 mg of zinc.*
